# Identification of minor effect QTLs for plant architecture related traits using super high density genotyping and large recombinant inbred population in maize (*Zea mays*)

**DOI:** 10.1186/s12870-018-1233-5

**Published:** 2018-01-18

**Authors:** Baobao Wang, Han Liu, Zhipeng Liu, Xiaomei Dong, Jinjie Guo, Wei Li, Jing Chen, Chi Gao, Yanbin Zhu, Xinmei Zheng, Zongliang Chen, Jian Chen, Weibin Song, Andrew Hauck, Jinsheng Lai

**Affiliations:** State Key Laboratory of Agrobiotechnology and National Maize Improvement Center, Department of Plant Genetics and Breeding, China Agricultural University, Beijing, China

**Keywords:** Bin map, Genotyping by sequencing (GBS), High resolution, Minor effect, Plant architecture, Quantitative trait loci (QTLs)

## Abstract

**Background:**

Plant Architecture Related Traits (PATs) are of great importance for maize breeding, and mainly controlled by minor effect quantitative trait loci (QTLs). However, cloning or even fine-mapping of minor effect QTLs is very difficult in maize. Theoretically, large population and high density genetic map can be helpful for increasing QTL mapping resolution and accuracy, but such a possibility have not been actually tested.

**Results:**

Here, we employed a genotyping-by-sequencing (GBS) strategy to construct a linkage map with 16,769 marker bins for 1021 recombinant inbred lines (RILs). Accurately mapping of well studied genes *P1*, *pl1* and *r1* underlying silk color demonstrated the map quality. After QTL analysis, a total of 51 loci were mapped for six PATs. Although all of them belong to minor effect alleles, the lengths of the QTL intervals, with a minimum and median of 1.03 and 3.40 Mb respectively, were remarkably reduced as compared with previous reports using smaller size of population or small number of markers. Several genes with known function in maize were shown to be overlapping with or close neighboring to these QTL peaks, including *na1*, *td1*, *d3* for plant height, *ra1* for tassel branch number, and *zfl2* for tassel length. To further confirm our mapping results, a plant height QTL, *qPH1a*, was verified by an introgression lines (ILs).

**Conclusions:**

We demonstrated a method for high resolution mapping of minor effect QTLs in maize, and the resulted comprehensive QTLs for PATs are valuable for maize molecular breeding in the future.

**Electronic supplementary material:**

The online version of this article (10.1186/s12870-018-1233-5) contains supplementary material, which is available to authorized users.

## Background

Plant architecture related traits (PATs) are of great importance for maize, as they are crucial indicators to the growth and development of the plant and finally influence the yield [1, 2]. Studies in past decades indicated that PATs are mainly affected by minor effect quantitative traits loci (QTLs), especially as reflected in the Nested Association Mapping (NAM) population [3–5]. Fine mapping coupled with further precise cloning of these QTLs would be of great value for maize breeding. Up to now, there were only a few plant architecture related QTLs had been cloned in maize, such as *tb1* [6], *qPH3.1* [7], and *qph1* [8]. However, they all are major effect QTL, with phenotype contribution larger than 10%. For minor effect QTL, the example of its cloning, even fine-mapping work has been rarely studied.

Previous QTL studies of agronomic traits in crops were mainly based on relatively small populations and a few hundred or less molecular markers. QTL intervals of these studies typically ranged from 2 to 50 cM [9–17], which was often hard to match physical map and also too large to locate candidate genes or causal polymorphisms. Small populations are easily leading to sampling bias (such as unintended assessment involved in individuals with extreme phenotype values), thus affecting the estimation of QTL effect and the accuracy of QTL location [18, 19], and unfavorable linkage event in small populations would even result in misleading inferences [20]. Besides, small populations always have the drawback of limited recombination thus limit the mapping resolution [19].

Marker density is another key consideration influencing the precision for recombinational breakpoints identification, and would also reduce the mapping resolution. Recent progress in high-throughput sequencing technologies has markedly enhanced and accelerated genotyping. The genotyping-by-sequencing (GBS) strategy, named “bin mapping”, has been successfully applied in rice [21], Arabidopsis [22], sorghum [23], soybean [24], and maize [25, 26], and re-mapped several genes or QTLs with known functions. However, almost all of these genes or QTLs verified were major effect ones. Minor effect QTLs were less investigated. And most of these studies were based on relatively small populations.

Several QTL mapping studies have been performed using large populations and increased marker density in targeted regions in plant. *ZmCCT*, an important photoperiod response gene in maize, was cloned by regional genotyping of key recombinants in 866 maize-teosinte BC_2_S_3_ lines and analysis of other diverse maize populations [27]. Two large RIL populations (1285 and 921 lines respectively) were used to localize an important QTL contributing to maize Sugarcane Mosaic Virus (SCMV) resistance, the key recombinants detected by Polymerase Chain Reaction (PCR) based markers could give a result of QTL interval small enough to identify candidate gene [28]. Recent bin mapping was conducted with deeply sequenced 132 RILs in rice, PCR genotyping of QTL intervals of an expanded population size (1709 lines) enabled the fine mapping of QTLs related to flowering time down to candidates including previously cloned gene [29]. These studies implied that combining large population with high density markers can be effective for QTL fine mapping. However, all these studies were performed for targeting local regions on major effect QTLs. Up to now, except the simulation, QTL studies conducted by actual data of large population (> 1000) and high density markers at the same time have not been reported.

In this work,we constructed a RIL population derived from Zhengdan958 (the most widely planted hybrid in China) of 1147 lines, generated a high density marker set using GBS strategy, constructed a bin map, and phenotyped the population in multiple environments. Fifty-one plant architecture related loci with small effect were mapped to relatively small confidence intervals, which led to a list of reasonable candidate genes. One of these QTLs was further verified with introgression lines. Thus we demonstrated a method for high resolution mapping of minor effect QTLs in maize, and the resulted comprehensive QTLs for PATs are valuable for maize molecular breeding in the future.

## Methods

### Plant materials and phenotypic evaluations

The RILs population used here includes 1147 lines, which was derived by crossing of Zheng58 and Chang7–2 (parents of the most widely planted maize hybrid Zhengdan958 in China recently [30], Zheng58 is a semi-Dent germplasm, which belongs to improved Reid heterotic group. While Chang7–2 is semi-Flint germplasm, which belongs to a Chinese native heterotic group, Tangsipingtou group), followed by single-seed descend process. F_7_ plants and the parental lines were phenotyped at three locations in China, Sanyuan (40°05′ N, 116°12′ E, 2010) and Shangzhuang (40°08′ N, 116°11′ E, 2011) in Beijing city, and Xinxiang (35°20′ N, 113°43′ E, 2012) in Henan Province. In Sanyuan, two replications and the randomized complete block design were applied, while for the remaining two environments, an augmented incomplete block design [5] with single replication was performed. Trials were performed with one row plots: row length of 3-m; row spacing of 0.5-m; and 13 plants per plot (80,000 plants per hectare) for all three locations. Traits of silk color (SC), ear height (EH), plant height (PH), tassel branch number (TBN), tassel length (TL), and upper leaf number (ULN), were measured on 10 (Sanyuan) or 5 (Shangzhuang and Xinxiang) plants per plot. PH and EH were collected at all three locations, while TL and TBN were only collected at Sanyuan and Xinxiang. SC and ULN were collected at Xinxiang only. SC was classified into five levels according to the intensity of red pigmentation. EH and PH were investigated as the distance from the node of the uppermost ear and the top of the main tassel spike to the soil surface, respectively. Relative ear height (EH/PH) was calculated as the ratio of EH to PH. TL was recorded as the length of the first branching node to the top of the main tassel spike. TBN was recorded as the number of primary tassel branches, and ULN was recorded as leaf numbers above the upper ear. Best Linear Unbiased Prediction (BLUP) was calculated for each phenotype by the *lme4* package [31] in R software across different environments, and used for subsequent analysis. The heritability estimates *h*^2^ were calculated by R software as reported formula [15].

An ILs population constructed by Chang7–2 as donor parent, Zheng58 as recurrent parent was used for verifying the reliability of *qPH1a*. In summer of 2011, 256 of BC_3_F_2_ or BC_4_F_1_ generation of ILs were planted in Shangzhuang in Beijing. Nineteen ILs (BC_4_F_1_) with remarkably higher PH were picked out and planted in Shangzhuang in summer of 2012. After checked by four Indel markers (Indel_1–87, Indel_1–89, Indel_1–91, Indel_1–97, Additional file 1: Table S8) that covering *qPH1a* interval, two of these ILs (BC_5_F_1_), 9_5 and 10_1, that harboring the *qPH1a* allele descend from Chang7–2 were found and crossed with Zheng58. In summer of 2013, the progenies (BC_6_F_1_) of 9_5 and 10_1 were phenotyped and genotyped, and 3 recombinants (BC_6_F_1_), G36–3, G40–7 and G41–8, were found and crossed with Zheng58 again. In the summer of 2014, progenies (BC_7_F_1_) of these 3 recombinants were planted in Shangzhuang, followed by measuring PH and genotyping by 4 Indel markers. A method named “recombinant-derived progeny testing strategy” [32] was invoked for inferring the location of *qPH1a*.

### DNA isolation, sequencing and SNP identification

Shoot tissues from five seedlings per genotype were bulked for total genomic DNA isolation and extraction using the CTAB protocol [33].

Zheng58 and Chang7–2 were deeply sequenced using Illumina HiSeq2000 for 28.41 (accession number, NCBI: SRX120186) and 24.95 (accession number, NCBI: SRX120903) fold coverage of the maize genome respectively [34]. DNA of 1130 RILs was processed according to the previously published GBS method [25, 35], in which ApeKI was used for genomic DNA digestion followed by ligation of the barcode adapter with variable length (see Additional file 1: Table S1) and a common adapter. Sets of 240 samples were pooled together and sequenced by Illumina HiSeq2000 platform to generate 100-nt paired-end reads.

SNP identification was conducted using a strict pipeline as reported [26], where BWA (version 0.5.9) [36] were used for read alignment and GATK (version 1.0.4418) were invoked for SNP identification [37]. For the RILs, raw reads were mapped to the position of SNPs retained from the two parents only, which reduced workload of the processing.

To ensure the map quality, only RILs with sequencing depth greater than 0.02× and genotyped SNPs more than 8000 were retained, which excluded 18 RILs from the dataset.

### Bin map construction and genetic properties analysis

The reported sliding window method [21] with minor modifications was adopted to conduct genotype calling for each RIL. (1) In each 15-SNPs window, the RIL genotype was defined by the rate of alleles from Zheng58 and Chang7–2: the window was called as homozygous if > 11/15 of the sites in the window were alleles from either of the parents or heterozygous otherwise. Based on the sliding window method, recombination breakpoints are observed as a region of several heterozygous, but do not tend to exceed more than six continuous windows.Therefore, we set heterozygous regions spanning less than seven uninterrupted windows as breakpoints and divided them into two by the midpoint. Then adjacent windows with the same genotype were merged together as a block. (2) Blocks with sequenced SNPs number less than 5 or physical length less than 300 kb were set to missing to avoid false double crossover. (3) Adjacent windows and successive small blocks with frequently transient genotypes were merged into a larger heterozygous block, and heterozygous block with SNP number less than 15 or physical length less than 1 Mb were set as missing to avoid false estimation.

The RIL population was further filtered by excluding the lines with greater than 15% residual heterozygous genotypes or with more than 360 breakpoints (91 RILs), to avoid material confounding, leaving a set of 1021 lines.Blocks without any breakpoints across the remaining population were grouped into bins, and bins with a physical length less than 5 kb were merged together. The allele frequency (Zheng58, Chang7–2 and heterozygous) of each bin was recorded to investigate instances of distorted segregation. Bins with a distorted segregation ratio larger than 2/1 or a heterozygous ratio larger than 15% were subsequently discarded (about 6% of total bins) for further improvement of the linkage map.QTL mapping software for RIL populations does not generally accept heterozygous loci, so bins with heterozygous genotype were regarded as missing followed by the “argmax” imputation method in R/qtl. Pair-wise recombinational fractions for all bins were calculated by the R/qtl function *est.rf*. The R/qtl function *est.map *(“kosambi” method) was manipulated for linkage map calculation. A genome wide recombination ratio analysis was calculated by (linkage distance)/(physical distance) in 1 Mb windows, and the filtered gene set gene density (B73 maize reference genome V2) and SNP density were recorded at the same time.

### QTL analysis

The QTL study of the Zhengdan958 RILs was conducted using the R/qtl package as reported [26]. Briefly, composite interval mapping (CIM) method was used for QTL identification, 1000 permutations test (*p* < 0.05) were used for defining QTL logarithm (base 10) of odds scores (LOD) threshold, 1.5 LOD-drop method was applied for defining QTL confidence interval, and linear QTL model was used for calculating QTL additive effect and phenotypic variation explained.

## Results

### Sequencing and SNP detection

Through deep sequencing of the two parents for above 24× coverage and identifying SNPs by a strict pipeline, 2,231,331 high-quality SNPs were detected between Zheng58 and Chang7–2. Approximately 67% of the SNPs were located in intergenic regions, 8% in exons, 12% in introns, and 14% within 2 kb upstream or downstream of genes (see Additional file 1: Figure S1).

1130 of the 1147 RILs were sequenced as described above, yielding about 1.8 billion 100-nt reads and an average of 0.08 × sequencing depth for each line (see Additional file 1: Figure S2). SNP calling generated 655,507 raw SNPs, with an average of 49,640 SNPs for each RIL and a mean of density of 1 SNP per 50 kb. While genotyping the RILs, we also genotyped the two parents using the GBS method for ~ 0.09× to estimate the error rate associated with RILs SNP detection. The error rates of genotyping Zheng58 and Chang7–2 were found to be 0.6% and 0.9%, respectively (see Additional file 1: Table S2). We examined these errors in 1 Mb windows and did not find obvious enrichment in any local regions, suggesting they can be easily excluded by the sliding window approach (see Additional file 1: Figure S3).

### Bin map construction

**Fig. 1:**
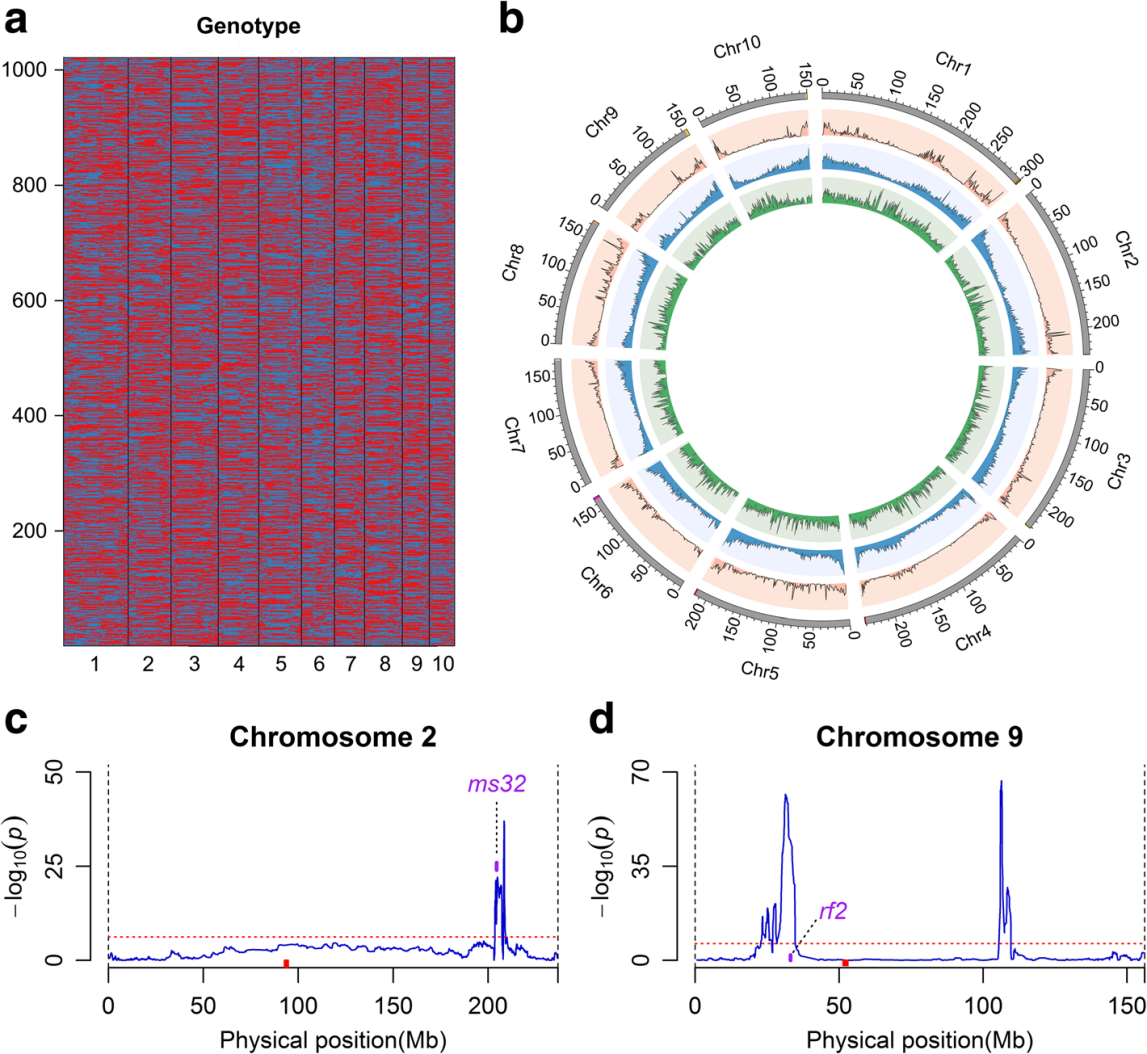
The genotype profile. **a** The graphic genotype of 1021 RILs. Red, Zheng58 genotype; Blue, Chang7–2 genotype.Heterozygouse genotypes were set as missing data and imputed by “argmax” method in R/qtl package. **b** Recombination ratio (outer, pink), gene density (middle, blue) and SNPs density (inner, green) distribution across the ten chromosomes. **c, d** The distribution of segregation distortions in chromosome 2 and 9. Segregation distortion was test by Chi-test and –log10 (P_Chi-test_) were plotted against its physical position. Candidate genes *ms32*, *rf2* were pointed out. Threshold of no distortion (*p* < 0.01, after Bonferroni-correction) were showed as red dashed lines

The modified sliding window method [21] was applied to construct the bin map for 1021 of the 1130 sequenced RILs, and 16,769 recombinant bins were identified across the genome, which presumably captures most of recombination events that took place in the process of population construction (Fig. 1a). The physical size of these bins range from 5 to 5.354 kb, with a mean of 126 kb (Table 1). 17 of 31 bins with size of more than 2 Mb were generated around 20 Mb regions flanking centromeres, where were always lacking of recombination. R/qtl [38] was adopted for linkage distance calculation, resulted in a genetic map of 2508.11 cM in total. The recombination rate was 1.227 cM/Mb, which was obviously lower than 6.95 cM/Mb of the IBM Syn10 DH population, which experienced ten generations of random mating [39].The largest interval between adjacent bins was 6.006 cM, while the average was 0.152 cM, revealing the high resolution of the bin map. Pair-wise recombinational fractions among bins were computed by *est.rf* in R/qtl and no obvious problems displayed (see Additional file 1: Figure S4).

**Table 1:** Summary of the bin map

Chr	Bins			Linkage(cM)			Rec^e^	Crossover
	NO.	Mean^a^	Max^b^	Total	Mean^c^	Max^d^		
1	2818	0.107	3.229	406.837	0.138	5.452	1.295	7.9
2	1794	0.132	5.354	238.895	0.131	4.378	0.990	4.7
3	1896	0.122	3.001	282.616	0.149	3.182	1.218	5.6
4	1624	0.149	2.841	221.605	0.137	5.148	0.919	4.4
5	2238	0.097	2.649	304.276	0.151	3.181	1.553	5.9
6	1322	0.128	4.656	206.735	0.156	5.697	1.224	4.1
7	1207	0.146	4.685	207.416	0.172	3.631	1.174	4.1
8	1559	0.113	4.999	265.544	0.163	4.088	1.449	5.2
9	1263	0.124	3.990	196.068	0.158	2.795	1.276	3.8
10	1048	0.143	4.479	178.120	0.167	6.006	1.167	3.5
Total	16,769	0.126	5.354	2508.111	0.152	6.006	1.227	4.9

^a,b^The average and maximal physical interval between the mid-point of adjacent bins, in Mb

^c,d^The average and maximal linkage interval of adjacent bins in cM

^e^Recombination ratio expressed as (length of linkage map)/(length of physical map), with a unit of cM/Mb

The 1021 RILs were produced from 6 generations of self pollination. Overall, theses RILs captured ~ 50,040 crossover events, resulting in an average of 4.9 per chromosome (Table 1), ranging from 3.5 for chromosome 10 to 7.9 for chromosome 1. We investigated the recombination ratio (expressed as the average linkage interval size across 1 Mb increments of the genome), the regional frequency of SNPs across the genome (SNP density), and the density of genes based on the filtered gene set (B73 reference genome V2) across the genome (gene density). The recombination ratio across the genome varied tremendously, ranging from 0 to 13.1 cM/Mb, with a mean of 1.227 cM/Mb, and was significantly correlated with gene density (*r* = 0.678, *p* < 2.20 E-16), but not overall SNP density (*r* = − 0.027, *p* = 0.248, Fig. 1b, Additional file 1: Table S3). The recombination ratio and gene density were reduced in regions surrounding centromeres and enriched near both telomeres on each chromosome (Fig. 1b), consisting with previous reports. Further analysis of the relationship between recombination and sub-regional SNP density, found that recombination ratio was positively correlated with SNP density in genic regions (exon, intron, 2 k–upstream and 2 k–downstream, *r* = 0.653, 0.503, 0.520, 0.415 respectively), but negatively correlated with intergenic regions (*r* = − 0.331, Additional file 1: Table S3). 149 regions with a 3 fold higher recombination ratio than the genomic average were identified and had an obviously elevated average gene density (37.7 for high recombination region comparing to 19.0 for genomic average) and genic region SNP density (see Additional file 2: Table S4). Consisting with previous reports that recombination events were preferentially occurring in gene regions or small multi-genic intervals [40–44]. Segregation distortion was observed in seven chromosomes (Chromosome 1, 2, 5, 6, 8, 9, 10, *p* < 0.01, Chi-square test, after Bonferroni-correction), including 1710 bins (10.2%), with total length of 155.8 Mb which covered 7.6% of the genome (see Additional file 1: Figure S5). Two of these regions contained gamete fertility related genes: *ms32* (*male sterility32*) [45] on chromosome 2 (Fig. 1c) and *rf2* (*restorer of fertility2*) [46] on chromosome 9 (Fig. 1d), suggesting a possibility that fertility related locus causing the distorted segregation in the RILs.

**Fig. 2:**
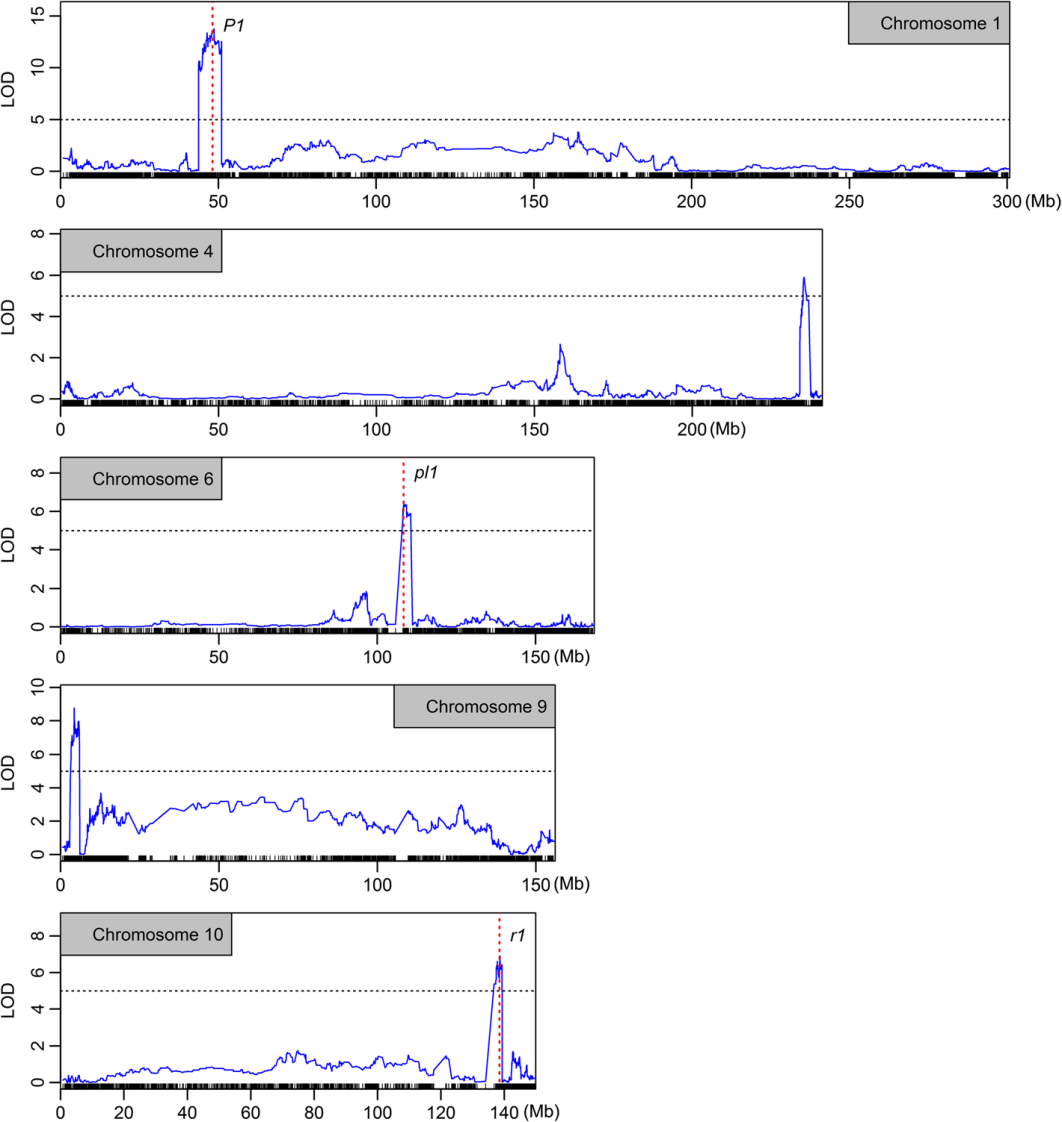
Five silk color QTLs and three putative candidate genes. The silk color QTLs located on chromosomes 1, 4, 6, 9 and 10 were plotted. The positions of candidate genes *P1*, *pl1* and *r1* were indicated by vertical dashed lines

The bin map quality and the QTL mapping pipeline were firstly tested using the silk color phenotype. The phenotypes were scored into 5 classes according to the intensity of red pigmentation (see Additional file 1: Figure S6). Totally, 5 QTLs were detected, located in chromosome 1, 4, 6, 9, 10 respectively. The most significant QTL (LOD = 13.76, Fig. 2) was located in the interval of 46.10–50.05 Mb on chromosome 1. The bin with the second highest LOD score was just overlapping with *P1* (*pericarp color1*) gene, which is known to contribute to the phlobaphene pigmentation of maize floral organs [47, 48]. The QTL on chromosome 6 was defined in the interval of 108.071–110.534 Mb with the peak just overlapping with *pl1* gene, which was contributed to purple pigment in maize plant [49, 50]. The chromosome 10 located QTL was in the interval of 137.325–139.207 Mb, with a bin with the second highest LOD score just encompassing *r1* gene, which was known contributed to silk color variation in maize [25, 34]. The other two QTLs may harbor genes with unknown function responsible for silk color controlling in maize. Mapping of the well studied *P1*, *pl1* and *r1* to relative small confidence intervals (3.950 Mb for *P1*, 2.463 Mb for *pl1* and 1.882 Mb for *r1*) demonstrated our map quality.

### QTL detection for six plant architecture related traits

The parents and RILs were phenotyped for traits of PH, EH, EH/PH, TBN, TL, and ULN. Zheng58 and Chang7–2 had large differences for all of these phenotypes, except for ULN. Transgressive segregation was observed for six PATs in the RILs population (see Additional file 1: Figure S7). Traits correlation coefficients were calculated (see Additional file 1: Table S5). As expected, PH and EH (*r* = 0.755), along with EH and EH/PH (*r* = 0.667) were strongly correlated, while a modest correlation was observed between PH and TL (*r* = 0.420), and ULN and EH/PH (*r* = − 0.360). The heritability estimates of PH, EH, EH/PH, TBN, and TL were high with all being greater than 0.7 (see Additional file 1: Table S6).

**Fig. 3:**
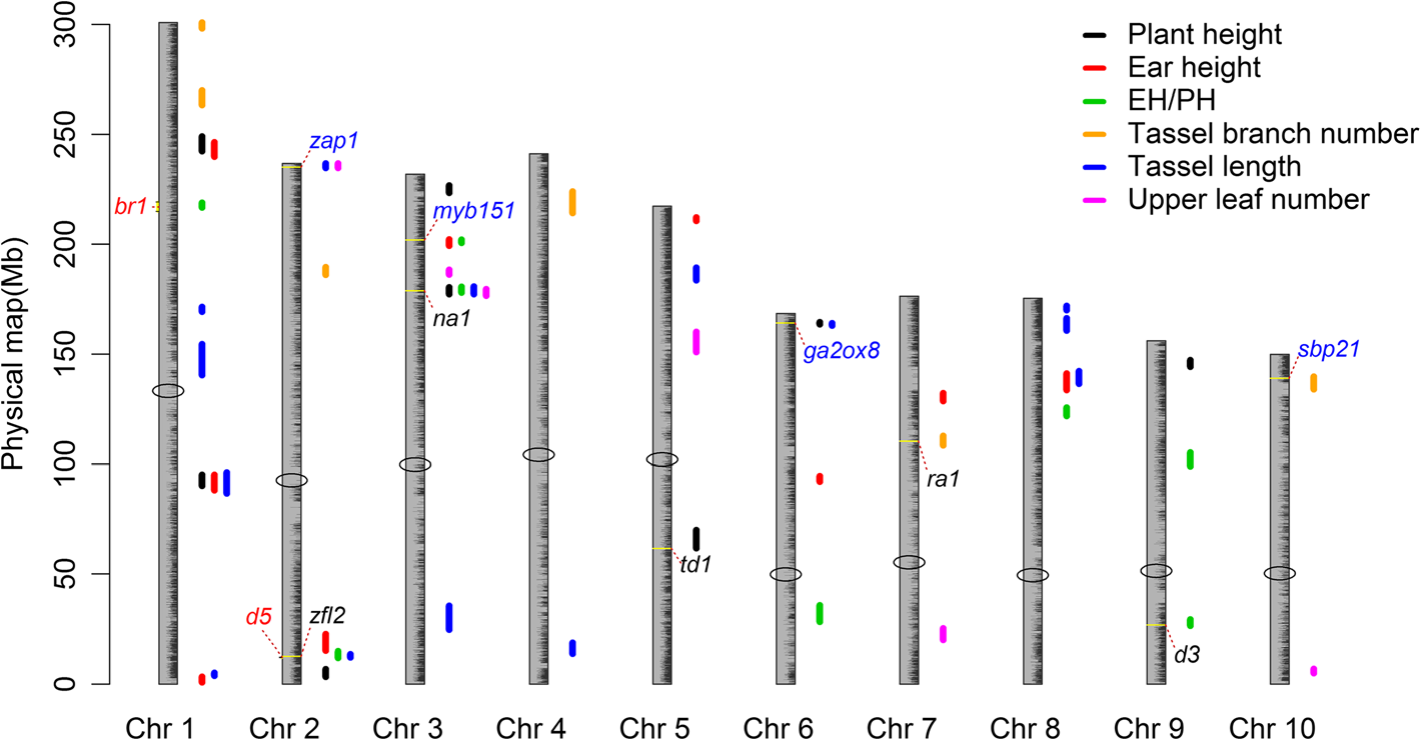
Fifty-one minor effect QTLs for 6 plant architecture traits. The ten chromosomes were displayed as grey bars according to the physical map, unit: Mb. Segments next to the chromosomes with different colors represented different QTLs for traits listed in the legend; Length of segments represented physical intervals of corresponding QTLs. Candidate genes were pointed out by short red dashed lines; red characters: reported mutants; blue characters: candidate genes inferred from homologues; black characters: genes with known function

Totally, 51 plant architecture related QTLs scattering across 10 maize chromosomes were detected above the threshold (*p* < 0.05, using 1000 times permutations), including 8 for PH, 9 for EH, 8 for EH/PH, 14 for TL, 6 for TBN, and 6 for ULN (Fig. 3, Additional file 1: Table S7). All of these QTLs were minor effect QTL, with explained phenotypic variation varied from 0.05% to 9.15%, a median of 2.01%. Seven chromosome regions, ranging in size from 2.12 to 9.53 Mb, contained QTLs (17/51 QTLs) for more than one traits, they were 86.73–96.26 Mb (*qPH1a*, *qEH1b*, *qTL1b*) and 239.92–249.10 Mb (*qPH1b*, *qEH1c*) on chromosome 1, 11.93–14.76 Mb (*qEP2*, *qTL2a*) and 234.72–236.84 Mb (*qTL2b*, *qULN2*) on chromosome 2, 176.73–180.82 Mb (*qPH3a*, *qEP3a*, *qTL3b*, *qULN3a*) and 199.25–202.15 Mb (*qEH3*, *qEP3b*) on chromosome 3, 133.70–142.27 Mb (*qEH8*, *qTL8a*) on chromosome 8. These regions could result from pleiotropy or linked genes. Thirty-one positive effect QTLs were inherited from Chang7–2.

Benefited from large sample size and saturated bin markers, most of the detected QTLs were defined into relatively small confidence intervals. Among the 51 QTLs, 32 were defined in intervals less than 5 Mb and 8 were located in less than 2 Mb, with a minimum of 1.03 Mb and a median interval size of 3.40 Mb for all QTLs (see Additional file 1: Table S7). Twenty-two of our QTLs overlapped with those identified in previously studies (Table 2), but all with intervals narrower in our study, which ranged from 1.04 to 9.72 Mb compared with other reports of intervals of 3 to 133 Mb [11–17, 51–61]. Six of them were overlapped with results from the large maize-teosinte backcross population, which had 1749 progenies but limited markers [51], with intervals ranging from 2.59 to 6.63 Mb in our RILs compared with corresponding intervals of 8–94 Mb. Four regions with closely linked QTLs for the same trait were determined (*qULN3a* and *qULN3b*, *qEP3a* and *qEP3b*, *qTL1c* and *qTL1d*, *qTL8a*, *qTL8b* and *qTL8c*), as adjacent two peaks were less than 25 Mb apart (Fig. 3, Additional file 1: Table S7), but could be clearly distinguished due to the superior resolution of our panel.

**Table 2:** Comparison of detected QTLs between this study and previous studies

QTL	Chr	Peak^a^	Int_Size^b^	Overlapped		Reference
				QTL^c^	Int^d^	
*qPH1a*	1	91.64	4.98	PLHT1.84w	56	Briggs et al., [51]
				phi001-umc67	129	Gonzalo et al., [12]
				Ph1a	39	Sibov et al., [16]
*qPH1b*	1	246.21	6.63	PLHT1.129w	25	Briggs et al., [51]
				qPH1b	25	Tang et al., [14]
				umc58-umc161a	88	Gonzalo et al., [12]
*qPH2*	2	4.86	3.38	bnl8.45a–bnlg381	27	Gonzalo et al., [12]
*qPH3a*	3	178.02	3.10	umc1167-umc1528	61	Salvi et al., [52]
				q3PH3	4	Ku et al., [53]
*qPH3b*	3	225.36	3.34	PLHT3.165w	8	Briggs et al., [51]
*qPH5*	5	62.64	8.31	qPH5a	30	Tang et al., [14]
				PH5	115	Samayoa et al., [54]
*qPH9*	9	146.60	2.91	phi017-csu093	133	Gonzalo et al., [12]
*qEH1b*	1	91.82	6.98	qEH1a	28	Park et al. [55]
				qEH-ln1b	118	Zheng & Liu, [13]
				qEH1.2	72	Tang et al., [56]
*qEH1c*	1	245.29	6.55	Qeh	69	Li et al., [57]
*qEH2*	2	16.82	7.32	bnlg1327-umc61	16	Nikolic et al., [58]
				qEH2–2-1	12	Wei et al., [17]
				qeh2a	8	Lima et al., [59]
*qEH3*	3	201.28	2.90	qeh3a	11	Lima et al., [59]
				qEH3.3	36	Tang et al., [56]
*qEH5*	5	211.49	1.57	Qqeh5–2	19	Zhang et al., [60]
*qEH6*	6	93.10	2.52	psr129b-M54/1C	–	Nikolic et al., [58]
				Qqeh6	17	Zhang et al., [60]
*qEH7*	7	130.24	3.59	Eh7b	34	Sibov et al., [16]
*qEH8*	8	140.23	7.44	EH8	27	Samayoa et al., [54]
*qTBN1a*	1	267.52	6.35	TBN1.150f	53	Briggs et al., [51]
*qTBN1b*	1	298.57	2.59	TBN1.196f	13	Briggs et al., [51]
*qTBN4*	4	218.10	9.72	qTBN4–2	12	Tang Hua et al., [61]
*qTBN7*	7	109.35	4.39	TBN7.43w	94	Briggs et al., [51]
				umc1695-ra1	107	Upadyayula et al., [11]
				phi034-ra1	93	Upadyayula et al., [15]
*qTL6*	6	163.42	1.04	U62-bmc1740	–	Upadyayula et al., [11]
				dup015-umc62	3	Upadyayula et al., [14]
*qULN3a*	3	178.17	2.95	umc1167-umc1528	61	Salvi et al., [52]
*qULN3b*	3	187.67	2.08	umc1167-umc1528	61	Salvi et al., [52]

^a^The physical position of QTL peaks detected in this study, unit: Mb

^b^The physical length of confident intervals for QTLs detected in this study, unit: Mb

^c^QTLs identified in previous studies

^d^The physical length of confident intervals for QTLs detected in previous studies. The intervals were calculated according to the physical position of flanking markers, which were got from MaizeGDB (http://www.maizegdb.org), unit: Mb

### Candidate genes analysis

With the aid of high resolution bin map and large RIL population, many QTLs were located to relatively small genomic intervals that included genes with known function (Fig. 3). For TBN, the QTL on chromosome 7 (*qTBN7*, LOD = 4.93, explained 7.78% phenotype variance) was located in an interval encompassing *ra1* (*ramosa1*) [62], an important gene known for maize floral organ branching. For PH and EH/PH, the QTLs (*qPH3a* and *qEP3a*, LOD = 6.74 and 5.14, 2.20% and 5.03% variance explained for PH and EH/PH respectively) in a ~ 3 Mb region of chromosome 3 includes *na1* (*nana plant1*) [63], an important gene in the brassinosteroid biosynthetic pathway that influences lower internodes length and affects maize height. The PH QTL on chromosome 5 (*qPH5*, LOD = 7.56, 3.05% variance explained) co-localized with *td1*, which was involved in CLAVATA signaling pathway and harboring known impact on plant height variation in maize [64]. The EH/PH QTL on chromosome 9 (*qEP9a*, LOD = 4.72, 1.72% variance expected) encompassing *d3*, a gene controls cytochrome P450-mediated biosynthesis of gibberellins that involved in maize dwarfing phenotype [65]. A TL QTL peak on chromosome 2 (*qTL2a*, LOD = 4.83, 1.99% variance expected) just overlapped with *zfl2*, which controls inflorescence development and architecture in maize [66]. An EH/PH QTL (*qEP1*, LOD = 11.80, 3.92% variance expected) in a 3.9 Mb region of chromosome 1 contains *br1* (*brachytic1*, http://www.maizegdb.org) [67], which has been reported to be associated with dwarfing as well. Another EH/PH QTL (*qEP2*, LOD = 3.88, 0.95% variance expected) in chromosome 2 co-localized with *d5* locus (*dwarf plant5*, http://www.maizegdb.org) [68], another important maize dwarf mutant.

In addition to genes that had been mapped or cloned in maize, several candidate genes inferred from homologues were also identified in our QTL intervals (Fig. 3). The largest ULN QTL, located on a tip of chromosome 2 harbored *zap1*, shown to be allelic to *ZmMADS3*, a transcription factor that influences maize internode number [69]. The EH and EH/PH co-localized QTL on chromosome 3, encompasses *myb151* which is homologous to *Arabidopsis AtMYB87*, the latter could suppress longitudinal elongation of multiple organs [70] that may contribute to plant dwarfing. The TBN QTL interval on chromosome 10 includes a gene, *sbp21*, homologous to *Arabidopsis AtSPL10*, which controls shoot-associated lateral organs development [71], may contribute to maize tassel branching. The QTL for PH that localized on chromosome 6, harbors a gene encoding a gibberellin 2-oxidase, which had been reported to participate in the pathway of gibberellin metabolism and may contribute to maize dwarfing [72]. Since the ultimate goal of QTL study is to probe causal genes or polymorphisms, the ability to map loci with a narrow confidence interval containing candidates indicated the strong power of the approach used in this study.

### Verification of *qPH1a* by introgression lines

**Fig. 4:**
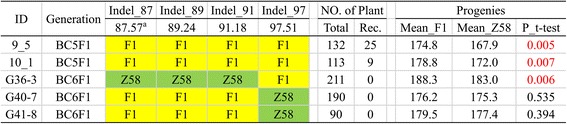
Verification of plant height QTL, *qPH1a*. Two BC_5_F_1_ ILs and three BC_6_F_1_ recombinants were genotyped by four Indels markers: Yellow, heterozygous; Green, Zheng58. The heterozygous segment of each ILs would segregate into two types of genotypes in its Zheng58-crossed progenies: homozygous Zheng58 and heterozygous. A simple t-test was performed to test PH difference between these genotypes. If significant difference (*p* < 0.05) was observed then *qPH1a* should be localized in the heterozygous region, else in the homozygous region. Finally, *qPH1a* was defined downstream of 91.18 Mb in chromosome 1. ^a^Physical position for Indels. Rec. number of recombinants found in these progenies

A population of ILs constructed by Chang7–2 as donor parent, Zheng58 as recurrent parent was used for verifying the reliability of *qPH1a*. In the summer of 2012, two ILs, named 9_5 and 10_1 (BC_5_F_1_), with heterozygous genotypes in the *qPH1a* region were chosen and backcrossed with Zheng58. In the summer of 2013, 132 and 113 progenies (BC_6_F_1_ generation ILs) of 9_5 and 10_1, were planted respectively, followed by measuring plant height and genotyping by 4 Indel markers (see Additional file 1: Table S8) that covering *qPH1a* interval. The progenies of these two ILs were segregated into two types of genotypes, heterozygous and homozygous Zheng58. The mean plant height of these two types of progenies (with genotype of homozygous Zheng58 or heterozygous) were significantly different (t-test, *p*-value = 0.005 and 0.007, Fig. 4), indicating that *qPH1a* was reliably located around its mapped interval on the bin map (Fig. 4, Chr1: 90.16–95.15 Mb). In the summer of 2014, three recombinants (BC_6_F_1_), named G36–3, G40–7 and G41–8, in the progenies of 9_5 and 10_1 were picked out and tested by the same method. G36–3 harbored heterozygous genotype upstream of Indel_91 and homozygous genotype downstream in the *qPH1a* region, while G40–7 and G41–8 just the opposite (Fig. 4). Analysis of progenies derive from G36–3 revealed that mean plant height of these two types of progenies corresponding to two segregated genotypes that downstream of Indel_91 (91.18 Mb) was significantly different (t-test, p-value = 0.006). Meanwhile, plant height of two types of progenies corresponding to two segregated genotypes upstream of Indel_91 was non-different (t-test, p-value = 0.535 and 0.494 respectively) as revealed by progenies of G40–7 and G41–8. For QTLs would only segregate in the heterozygous regions, thus *qPH1a* was further confirmed to be located downstream of 91.18 Mb, given its further interval of 91.18–95.15 Mb on chromosome 1.

## Discussion

### Accurately mapping of minor effect QTLs by combining large population and super-high density bin map

Large amount of resources had been used to study agronomic QTLs in staple crops, however, relatively few underlying genes and regulatory elements have been identified. Previous reports revealed that enlarging population size could effectively enhance the efficiency of QTL mapping, and reduce the false-positive rate caused by small populations [18, 19]. Besides, the utility of high-density markers was another efficient way to improve the QTL mapping resolution [21]. However, for precisely location of minor effect QTLs, large population or high density marker alone is not enough [51, 73]. Here, combining a panel of > 1000 RILs and GBS-based high density markers resulted in a bin map with an average physical / linkage interval of 126 kb / 0.152 cM (Table 1). This resolution is greatly improved than previously linkage maps constructed with traditional molecular markers in maize which ranged from 7 to 24 cM for the average marker intervals [9–17]. Among the QTLs detected, 63% (32 of 51) were defined in intervals less than 5 Mb, 8 were even located in less than 2 Mb, with a median interval size of 3.40 Mb. These intervals identified were relatively small for minor effect QTLs [11–17, 51–61]. Comparing with overlapped QTLs obtained in other studies using small population sizes or sparse marker coverage found that, QTL intervals detected here were narrowed by 1.1 to 45.7 fold (Table 2) [11–17, 51–61].

Closely linked loci in coupling phase or repulsion phase can cause misevaluation or misdetection of QTLs, which is a major challenge in quantitative trait studies. In our study, there were 4 regions containing closely linked QTLs for the same trait (as adjacent two peaks were less than 25 Mb apart) (Fig. 3, Additional file 1: Table S7), but could be clearly distinguished between them due to the high marker density and large population. On chromosome 3, two closely linked QTLs for ULN with different effects (*qULN3a* and *qULN3b*) were identified in the region where only a single QTL were detected by Salvi et al. [52] (Table 2). These results clearly demonstrate the power of combining large population and high density markers.

Besides the increased resolution, several genes with known function were located in overlapping or close neighboring QTL peaks. For silk color, the QTL peak in chromosome 6 was just overlapping with *pl1*, and QTLs in chromosome 1 and 10 was mapped to *P1* and *r1* that both overlapping with bins with the second highest LOD scores. For agronomic traits, which were more complex than silk color, our data also has outstanding powers, as suggested by the QTL peak of *qTL2a* overlapped with *zfl2*, peak of *qEP3a* and *qEP9a* located just 23.9 and 53.6 kb away from *na1* and *d3* respectively. This indicated that even minor effect loci for a quantitative trait could be fine mapped.

In summary, we have demonstrated that high resolution QTL mapping can be achieved using a large RIL population together with super high density markers. However, it is worth to note that there are still some limitations if only one bi-parental population is used. For example, crop genomes like maize are often high diverse, such that two parental lines can only capture small part of overall genome diversity. For any given one bi-parental population, many QTLs will likely be missed due to lack of polymorphism between the two parental lines. Therefore, for any specific traits, it is very important to consider mapping QTLs with population of multiple parental lines.

### Minor effect QTLs are important for modern maize breeding

The breeding of maize is the process of pyramiding favorable alleles. It’s known that important genes or major QTLs for agronomic trait, such as *tb1* [74], *tga1* [75], were selected and even potentially fixed in the process of maize domestication or early breeding. Thus the subsequent commercial breeding may rely more on minor effect QTLs. The minor effect QTLs mapped for flowering time [76], leaf architecture [5], inflorescence architecture [3] and height related traits [4] in NAM population are good examples. In this study, by combining large population and high-density bin map, we mapped a list of QTLs for PATs, with all of them having minor effects. These QTLs were segregating between two parental lines of the widely adopted Chinese maize hybrid, suggesting that parental lines of modern commercial hybrid can often differ in minor effect QTLs with majority of major effect QTLs fixed.

Meanwhile, our results indicated that several genes for plant architecture are likely candidate genes, such as *an1*, *td1*, *d3*, and *ra1*. The mutation of these genes would cause extreme change in corresponding phenotypes, but were defined as minor effect QTLs in our results. It was reported that mild alleles with mutations in gene body (such as *qph1* [8]) or regulatory region (such as *OsLG1* [77]) of important genes in maize and rice could cause QTL-like minor effect for the same phenotype and could be practically used in breeding. The minor effect QTLs identified in this study probably act in the same way and could be crucially useful for breeding, even though their exactly causal variations are still unknown.

Additionally, Zhengdan958 is the most widely used maize hybrid in China. Their two parental lines, Zheng58 and Chang7–2, are the top breeding materials in our current breeding system. Zheng58, an improved Reid germplasm, is derived from 5003, which was derived from an elite hybrid from the US and have being the most important dwarf germplasm in China [78]. Chang7–2 is a classic native inbred from which more than 200 lines were derived and many of these lines were utilized as parental lines of popular hybrid varieties used in China [79]. We believe that the QTLs identified from these two top elite lines, which were derived from the US and native Chinese germplasm respectively, would be of great value for maize breeding in China and even other regions of the world in the future.

## Conclusions

In this study, by combining large population and high-density bin map, we mapped a list of minor effect QTLs for PATs in maize. The interval sizes of these QTLs were greatly reduced as compared with previous studies using smaller size of population or smaller number of markers. A number of well studied genes with similar function were located in these QTL intervals, and a plant height QTL were further confirmed by an introgression lines. These QTLs represent a set of reliable resource for maize molecular breeding in the future, and the method used here demonstrated the power of combining large population with high-density markers for minor effect QTL mapping.

## Additional files

Additional file 1: Figure S1. Distribution of SNPs polymorphic between Zheng58 and Chang7–2 in different genomic regions. **Figure S2.** Sequencing depth profile of Zhengdan958 RILs. **Figure S3.** Distribution of false GBS SNPs in 1 Mb windows by parent. **Figure S4.** Pair-wise recombinational fractions (upper left) and LOD scores (lower right) of the bins. **Figure S5.** The distribution of segregation distortions across ten chromosomes.Segregation distortions were tested by Chi-test, and –log_10_ (P_Chi-test_) were plotted against their physical positions. Threshold of no distortion (*p* < 0.01, after Bonferroni-correction) were showed as red dashed lines. **Figure S6.** The 5 classes of silk color. **Figure S7.** Phenotypic distributions of PH, EH, EH/PH, TNB, TL and ULN. **Table S1.** The 240 barcodes used in GBS. **Table S2.** GBS error rate for parental lines. **Table S3.** Pair-wise Pearson’s correlation coefficients (lower left) and *p*-values (upper right) among recombination, gene density, and SNP density in 1 Mb intervals by genomic region. **Table S5.** Pearson correlation coefficients among different traits in two environments.Lower left, the correlation coefficients; Upper right, p-values of correlation test. **Table S6.** Heritability of different traits. **Table S7.** Summary of mapped QTLs. **Table S8.** Markers used for verifying *qPH1a*. (PDF 934 kb)

Additionnal file 2: Table S4. Regions with 3 fold higher recombinant ratio than genome average. D_Downstream, D_Exon, D_Intron, D_Upstream, D_Intergenic: SNP density in 2 k–downstream, exon, intron, 2 k–upstream of genic regions and SNP density in intergenic regions. (XLSX 21 kb)

## Abbreviations

BLUP: Best linear unbiased prediction
CIM: Composite interval mapping
EH: Ear height
GBS: Genotyping-by-sequencing
ILs: Introgression lines
LOD: Logarithm (base 10) of odds scores
NAM: Nested association mapping population
PATs: Plant architecture related traits
PCR: Polymerase chain reaction
PH: Plant height
QTL: Quantitative trait locus
RIL: Recombinant inbred lines
SC: Silk color
SCMV: Sugarcane mosaic virus
TBN: Tassel branch number
TL: Tassel length
ULN: Upper leaf number
