# Supplementary material for: Identification of minor effect QTLs for plant architecture related traits using super high density genotyping and large recombinant inbred population in maize (Zea mays)
Source: BMC Plant Biol. 2018 Jan 18;18:17. doi: 10.1186/s12870-018-1233-5 (PMC5774087; doi:10.1186/s12870-018-1233-5)
Supplement: Supplementary file 1 — Distribution of SNPs polymorphic between Zheng58 and Chang7–2 in different genomic regions. Figure S2. Sequencing depth profile of Zhengdan958 RILs. Figure S3. Distribution of false GBS SNPs in 1 Mb windows by parent. Figure S4. Pair-wise recombinational fractions (upper left) and LOD scores (lower right) of the bins. Figure S5. The distribution of segregation distortions across ten chromosomes.Segregation distortions were tested by Chi-test, and –log10 (PChi-test) were plotted against their physical positions. Threshold of no distortion (p < 0.01, after Bonferroni-correction) were showed as red dashed lines. Figure S6. The 5 classes of silk color. Figure S7. Phenotypic distributions of PH, EH, EH/PH, TNB, TL and ULN. Table S1. The 240 barcodes used in GBS. Table S2. GBS error rate for parental lines. Table S3. Pair-wise Pearson’s correlation coefficients (lower left) and p-values (upper right) among recombination, gene density, and SNP density in 1 Mb intervals by genomic region. Table S5. Pearson correlation coefficients among different traits in two environments.Lower left, the correlation coefficients; Upper right, p-values of correlation test. Table S6. Heritability of different traits. Table S7. Summary of mapped QTLs. Table S8. Markers used for verifying qPH1a. (PDF 934 kb) [file 12870_2018_1233_MOESM1_ESM.pdf]

Figure. S1. Distribution of SNPs polymorphic between Zheng58 and Chang7-2 in different genomic regions.

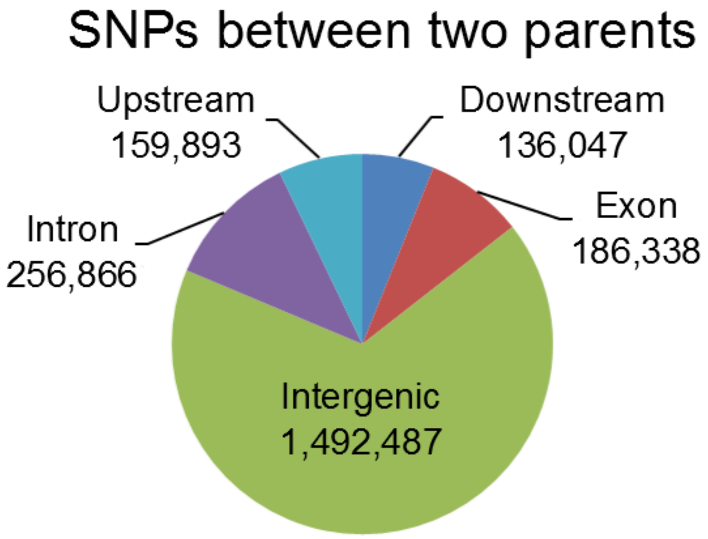

Figure. S2. Sequencing depth profile of Zhengdan958 RILs.

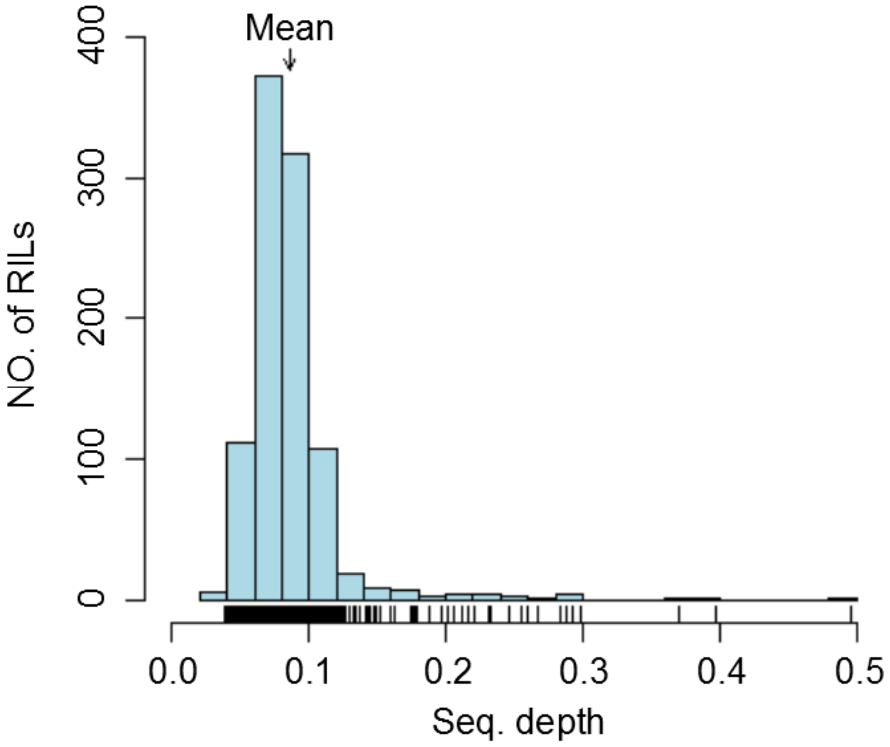

Figure. S3. Distribution of false GBS SNPs in 1 Mb windows by parent.

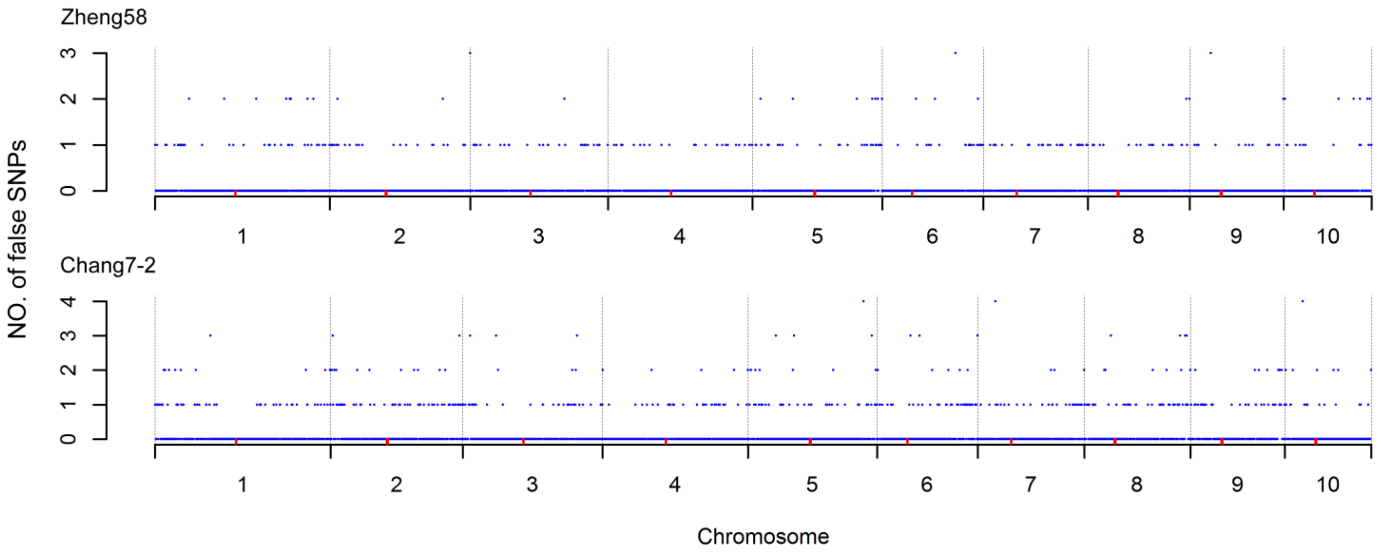

Figure S4. Pair-wise recombinational fractions (upper left) and LOD scores (lower right) of the bins.

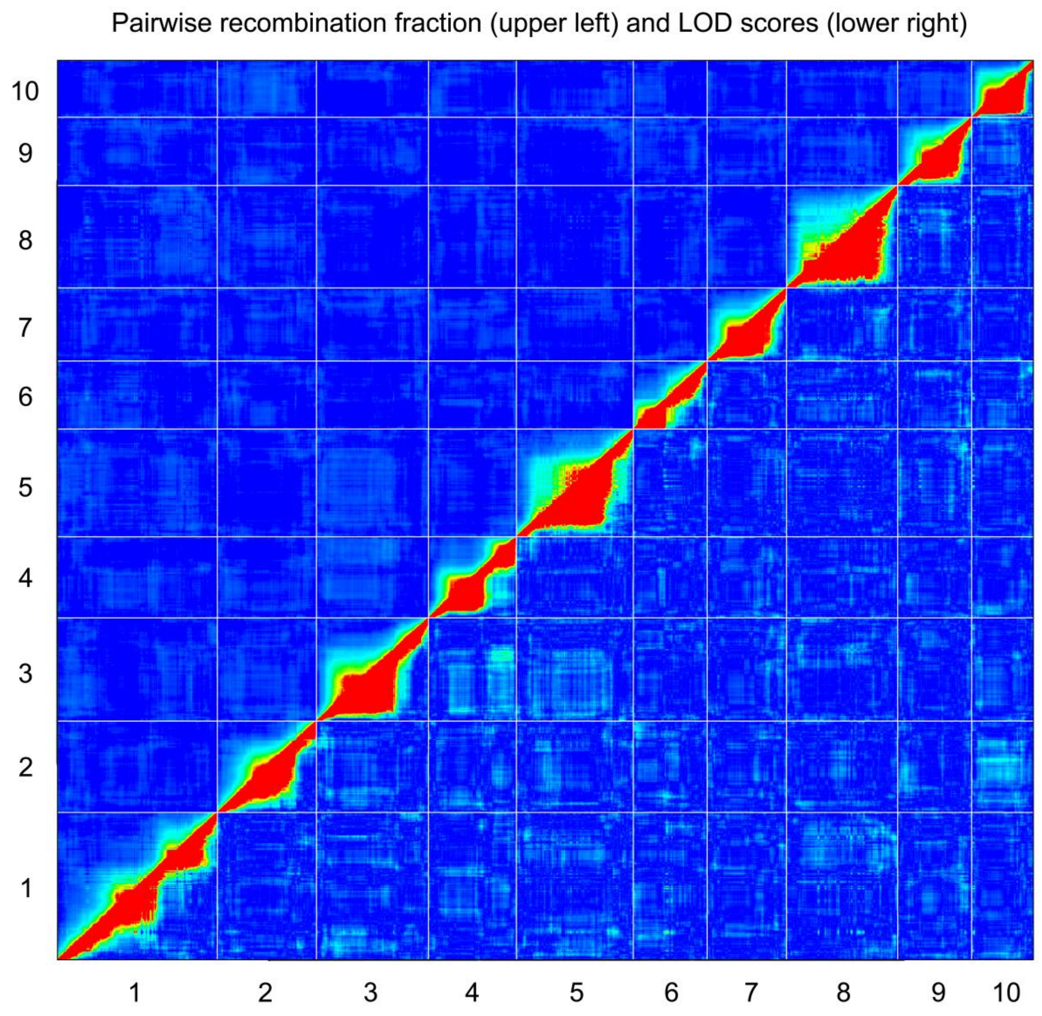

Figure. S5. The distribution of segregation distortions across ten chromosomes. Segregation distortions were tested by Chi-test, and –log10 ($P_{Chi\text{-}test}$) were plotted against their physical positions. Threshold of no distortion ($p < 0.01$, after Bonferroni-correction) were showed as red dashed lines.

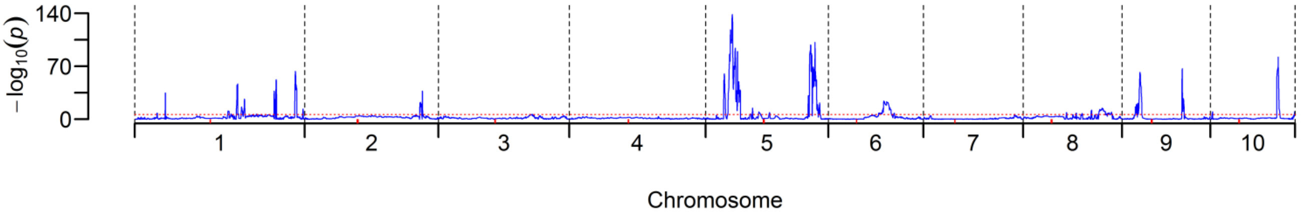

Figure. S6. The 5 classes of silk color.

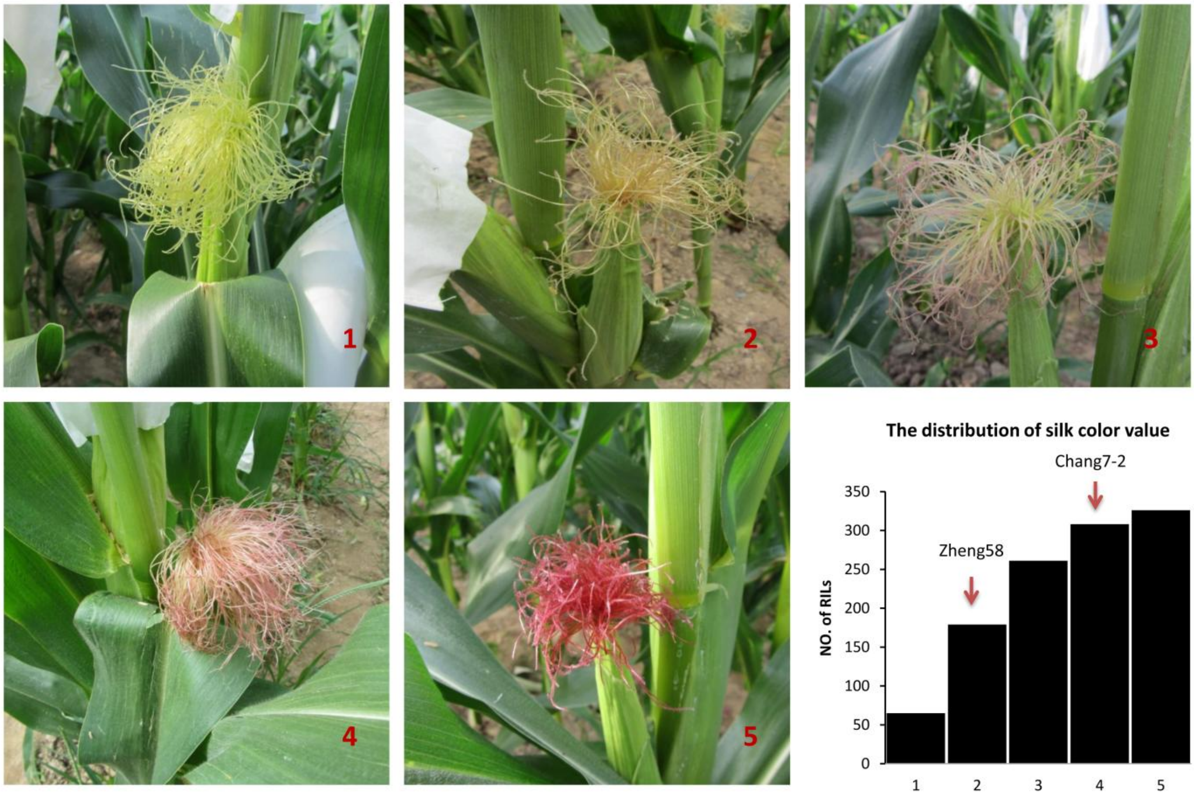

Figure. S7. Phenotypic distributions of PH, EH, EH/PH, TNB, TL and ULN.

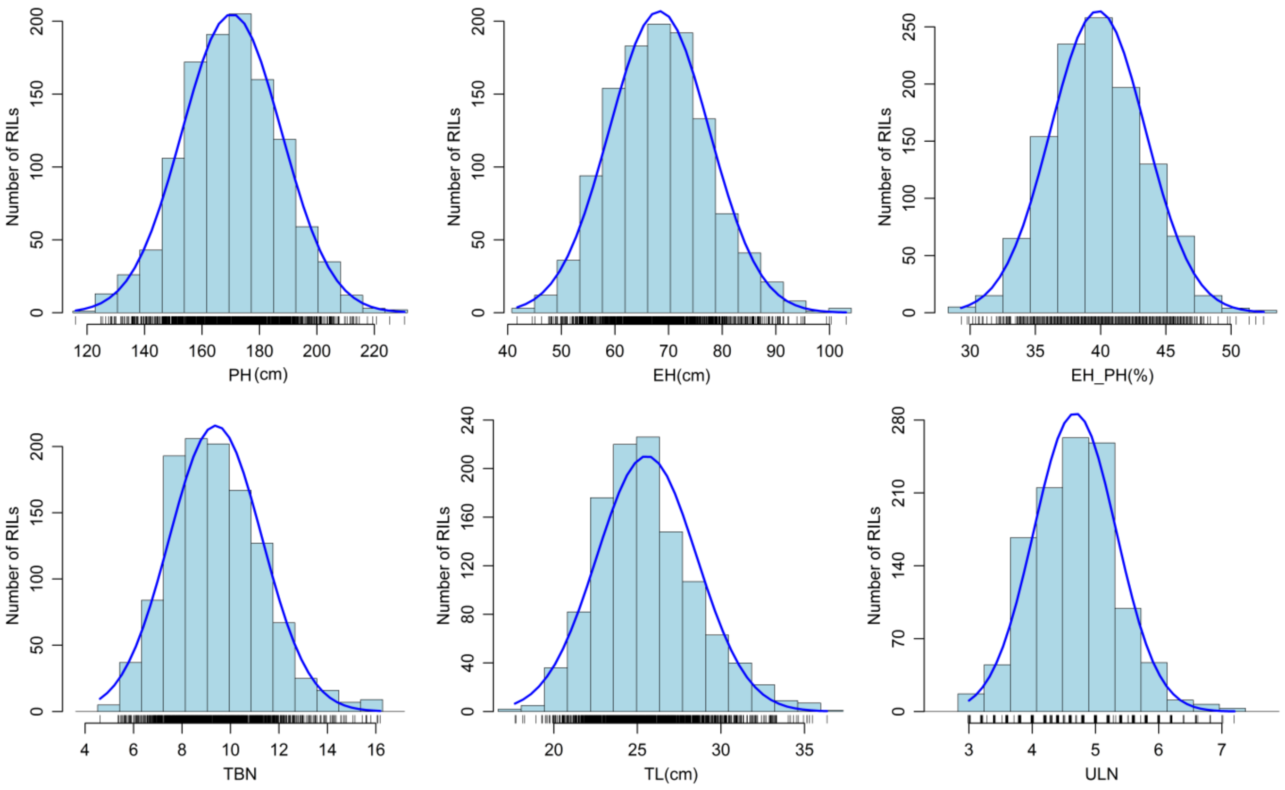

Table S1. The 240 barcodes used in GBS

ID	Barcodes	ID	Barcodes	ID	Barcodes	ID	Barcodes	ID	Barcodes	ID	Barcodes
1	TGCA	41	GCCAGT	81	GTTAGC	121	TGCATAT	161	CAGTGAC	201	AACTAGGA
2	ACTA	42	GGAAGA	82	AGCATT	122	AGTTCGA	162	GCACACT	202	ACAATGGA
3	CAGA	43	GTACTT	83	GATGTC	123	ACGCTGA	163	TGTATCA	203	GATCGTGT
4	AACT	44	GTTGAA	84	TGTTAC	124	TAAGCTT	164	CAAGATC	204	CAATGAGA
5	GCGT	45	TAACGA	85	TGACCT	125	TGATACA	165	TATTGCA	205	TACGGTGT
6	GTAA	46	TGGCTA	86	TTGCAC	126	ACCGAGT	166	GGATATC	206	GCATAAGA
7	GATC	47	TATTTTT	87	TAGTGC	127	ATGTCAA	167	CCGAGCT	207	GCCGCAGT
8	TCAC	48	CTTGCTT	88	TGAATC	128	GGTGGCA	168	ATCTAGC	208	TTGCGTGA
9	TGCGA	49	ATGAAAC	89	CGCTCA	129	TCGTTAA	169	CGGCTCA	209	ACAGATGA
10	CGCTT	50	AAAAGTT	90	ACGCTC	130	TGATCAT	170	GGTACAC	210	CAGATAGA
11	TCACC	51	GAATTCA	91	CTGATC	131	TAGGTCA	171	TCATGAC	211	TGACGTGT
12	CTAGC	52	GAACTTC	92	TACGCC	132	GCAACGT	172	TTCGGAC	212	CTCGCGGA
13	ACAAA	53	GGACCTA	93	CCGTAC	133	ATAGTCA	173	GCTTAGC	213	GTGCATGT
14	AGCCC	54	GTCGATT	94	TCGACC	134	TAGCCAT	174	ACTGCCA	214	AATACGGA
15	GTATT	55	AACGCCT	95	GCATCC	135	AGGAGTC	175	TAGTACT	215	AGCGGCGT
16	CTGTA	56	ACGTGTT	96	CGTACC	136	GTAGAGC	176	GACACTC	216	TAAGCAGA
17	ACCGT	57	ATTAATT	97	TTCAAGT	137	GACCTAT	177	CGAGTAC	217	CGTAGTGT
18	GCTTA	58	ATTGGAT	98	GAGCAGT	138	ATTAGCA	178	ATATGTC	218	ACGTTGGT
19	GGTGT	59	CATAAGT	99	ACACGGT	139	TGTCATT	179	TGGACCT	219	GTCAAGAA
20	AGGAT	60	CGCTGAT	100	GACGTGA	140	GCCAGAT	180	TTCGCCA	220	CGGTTAGT
21	ATTGA	61	CGGTAGA	101	TCATAGT	141	CCAGGTA	181	AGTTACT	221	TCTATGTA
22	CATCT	62	CTACGGA	102	TTACGAT	142	TGGCAAC	182	GTGCCAC	222	CGGCGGAT
23	CCTAC	63	GCGGAAT	103	TACGGTA	143	ATGATCT	183	ACCGGTC	223	CCACACGT
24	GAGGA	64	TAGCGGA	104	CGTGAAT	144	CATGTTA	184	TATAGTC	224	GACAGAAT
25	GGAAC	65	TCGAAGA	105	AACTTGT	145	TGTAAGC	185	ACTCCGC	225	TTGTCGGA
26	TAATA	66	TGCTGGA	106	GCTATAA	146	GTGTACA	186	GTATTAC	226	GGACAATA
27	TACAT	67	ACGACTAC	107	CTTGAGA	147	GATAGCT	187	TGAGCGC	227	ACTGGTGT
28	GGTTGT	68	TAGCATGC	108	GATCATA	148	TACGAGC	188	GAGTCGC	228	TACAGGAA
29	TTCAGA	69	TAGGCCAT	109	CAGGTAT	149	TTGACTA	189	TCCACGC	229	AAGAACGT
30	TAGGAA	70	TGGTACGT	110	TGCAATA	150	ATACTGC	190	GTTATTC	230	CTAGGTGT
31	GCTCTA	71	TCTCAGTC	111	ATATCGT	151	GCGGCTA	191	TATGTAC	231	CGTAAGAA
32	CCACAA	72	CCGGATAT	112	AGTCTAT	152	TTAGACT	192	AGACAAGT	232	GTGTCAGT
33	CTTCCA	73	CGCCTTAT	113	GTCTGAA	153	AGGTTAC	193	GGCCGAGT	233	AGACTGAA
34	GAGATA	74	ACAGGGAA	114	TGTGCAA	154	GGCAACT	194	ATACAGGA	234	TCTTAAGT
35	ATGCCT	75	ACGTGGTA	115	CGACAGT	155	TCACCGA	195	ACAAGAGT	235	GAACGTAA
36	AGTGGA	76	CCATGGGT	116	GATGCAT	156	AAGGTTC	196	GACAATGA	236	TCAGTGGT
37	ACCTAA	77	CGCGGAGA	117	CTAATGT	157	GAAGTCAA	197	CGGCTGGA	237	CCACCTGA
38	ATATGT	78	CGTGTGGT	118	AGCGTTA	158	TAATTGC	198	CAAGAAGT	238	GATAGCAA
39	ATCGTA	79	GCTGTGGA	119	TCAGTAT	159	GCTGGAC	199	GAACTAGA	239	TGTGCAGT
40	CGCGGT	80	GGATTGGT	120	GTTACGA	160	GTCATCA	200	TCGAGTGT	240	GTGAGGGT

Table S2. GBS error rate for parental lines.

Parents	Sequencing depth	Total SNPs	Error SNPs
Zheng58	0.085×	50999	285(0.6%)
Chang7-2	0.089×	52491	493(0.9%)

Table S3. Pair-wise Pearson's correlation coefficients (lower left) and p-values (upper right) among recombination, gene density, and SNP density in 1Mb intervals by genomic region.

		Recombination	Gene_density	SNPs_density(NO./Mb)					
		Unit: cM/Mb	Unit: NO./Mb	Overall	Downstream	Exon	Intron	Upstream	Intergenic
Recombination			<2.20e-16	0.248	<2.20e-16	<2.20e-16	<2.20e-16	<2.20e-16	5.31E-47
Gene_density		0.678		0.004	<2.20e-16	<2.20e-16	<2.20e-16	<2.20e-16	2.65E-52
SNPs_density	Overall	-0.027	0.063		<2.20e-16	<2.20e-16	<2.20e-16	<2.20e-16	<2.20e-16
	Downstream	0.415	0.599	0.504		<2.20e-16	<2.20e-16	<2.20e-16	1.64E-09
	Exon	0.653	0.819	0.298	0.73		<2.20e-16	<2.20e-16	3.45E-12
	Intron	0.503	0.63	0.396	0.638	0.792		<2.20e-16	0.122
	Upstream	0.52	0.715	0.462	0.789	0.836	0.706		0.024
	Intergenic	-0.331	-0.326	0.885	0.132	-0.153	-0.034	0.05	

Table S5. Pearson correlation coefficients among different traits in two environments. Lower left, the correlation coefficients; Upper right, p-values of correlation test.

	PH	EH	EH_PH	TBN	TL	ULN
PH		<2.200E-16	0.398	0.001	<2.200E-16	<2.200E-16
EH	0.755		<2.200E-16	2.60E-11	0.047	0.197
EH_PH	0.025	0.667		4.90E-11	<2.200E-16	<2.200E-16
TBN	0.098	0.195	0.193		0.007	0.803
TL	0.420	0.059	-0.376	-0.08		1.24E-07
ULN	0.281	-0.038	-0.360	0.007	0.156	

Table S6. Heritability of different traits.

Traits	Heritability
PH	0.857
EH	0.835
EH_PH	0.808
TBN	0.744
TL	0.776

Table S7. Summary of mapped QTLs.

QTL	Chr	Peak_Bin	Pos(Mb)	Interval(cM)	Interval(Mb)	Lod	Var(%)	Add	Positive	Candidate_gene
qPH1a	1	Bin1_994	91.64	159.72-162.53	90.16-95.15	21.83	9.15	9.75	Chang7-2	
qPH1b	1	Bin1_2080	246.21	284.73-293.28	242.47-249.10	5.88	2.47	5.40	Chang7-2	
qPH2	2	Bin2_95	4.86	14.37-29.67	3.35-6.73	3.90	1.38	3.67	Chang7-2	
qPH3a	3	Bin3_1242	178.02	161.99-170.48	177.41-180.51	6.74	2.20	-5.47	Zheng58	na1
qPH3b	3	Bin3_1807	225.36	253.18-264.26	223.42-226.75	4.43	1.28	-4.13	Zheng58	
qPH5	5	Bin5_805	62.64	95.85-112.52	61.69-70.00	7.56	3.05	6.00	Chang7-2	td1
qPH6	6	Bin6_1244	163.98	186.49-193.06	163.65-164.68	7.62	2.60	-5.90	Zheng58	GRMZM2G155686
qPH9	9	Bin9_1138	146.60	153.58-165.83	144.34-147.25	4.67	1.48	4.27	Chang7-2	
qEH1a	1	Bin1_12	2.95	0.00-4.56	0.85-3.37	3.71	0.95	-1.89	Zheng58	
qEH1b	1	Bin1_995	91.82	158.39-162.53	88.17-95.15	15.34	7.28	4.57	Chang7-2	
qEH1c	1	Bin1_2069	245.29	284.09-289.66	239.92-246.47	14.68	6.11	4.75	Chang7-2	
qEH2	2	Bin2_284	16.82	57.30-75.50	15.28-22.59	3.45	0.81	-2.11	Zheng58	
qEH3	3	Bin3_1537	201.28	199.17-204.9	199.25-202.15	4.12	1.51	2.44	Chang7-2	GRMZM2G167829
qEH5	5	Bin5_2106	211.49	271.52-280.17	210.65-212.22	4.98	1.63	2.65	Chang7-2	
qEH6	6	Bin6_457	93.10	50.90-55.64	92.08-94.59	7.70	3.32	3.55	Chang7-2	
qEH7	7	Bin7_757	130.24	96.50-112.88	128.70-132.29	4.87	2.10	-2.63	Zheng58	
qEH8	8	Bin8_1065	140.23	155.35-163.79	133.70-141.15	5.42	1.71	2.53	Chang7-2	
qEP1	1	Bin1_1835	217.16	274.25-276.42	216.69-218.90	11.80	3.92	0.015	Chang7-2	br1
qEP2	2	Bin2_237	13.74	45.75-56.27	11.93-14.76	3.88	0.95	-0.009	Zheng58	d5
qEP3a	3	Bin3_1255	178.84	165.64-171.12	178.15-180.78	5.14	5.03	0.011	Chang7-2	na1
qEP3b	3	Bin3_1536	201.25	200.60-204.56	200.56-202.05	9.97	4.07	0.016	Chang7-2	
qEP6	6	Bin6_153	33.72	23.85-27.50	28.14-35.72	5.68	2.34	0.012	Chang7-2	
qEP8	8	Bin8_868	122.93	127.68-136.21	121.90-125.66	6.79	2.92	0.013	Chang7-2	
qEP9a	9	Bin9_321	26.64	67.69-77.51	26.33-29.20	4.72	1.72	-0.007	Zheng58	d3
qEP9b	9	Bin9_679	101.10	95.05-99.34	98.98-105.28	4.03	0.22	-0.005	Zheng58	
qTBN1a	1	Bin1_2357	267.52	323.03-333.30	263.50-269.85	5.67	3.21	0.60	Chang7-2	
qTBN1b	1	Bin1_2798	298.57	396.41-406.84	298.23-300.82	4.06	1.37	0.49	Chang7-2	
qTBN2	2	Bin2_1205	187.94	145.42-150.49	186.05-189.50	4.44	1.69	0.45	Chang7-2	
qTBN4	4	Bin4_1372	218.10	171.31-176.36	214.23-223.96	5.84	2.80	0.58	Chang7-2	
qTBN7	7	Bin7_579	109.35	82.85-86.57	108.42-112.82	4.93	7.78	1.05	Chang7-2	ra1
qTBN10	10	Bin10_844	138.85	107.90-132.94	134.08-139.74	3.82	1.02	0.39	Chang7-2	GRMZM2G148467
qTL1a	1	Bin1_55	5.01	7.94-14.60	3.81-5.05	4.36	2.33	-0.81	Zheng58	
qTL1b	1	Bin1_996	93.49	156.70-163.08	86.73-96.26	5.06	1.52	0.33	Chang7-2	
qTL1c	1	Bin1_1218	150.75	172.24-175.76	140.54-154.58	5.23	0.21	0.21	Chang7-2	
qTL1d	1	Bin1_1357	170.14	190.63-194.20	169.43-171.52	4.67	0.05	0.47	Chang7-2	
qTL2a	2	Bin2_220	12.64	46.64-51.68	12.11-13.57	4.83	1.99	0.89	Chang7-2	zfl2
qTL2b	2	Bin2_1783	236.11	233.18-238.90	234.72-236.84	6.63	1.81	0.85	Chang7-2	zap1
qTL3a	3	Bin3_348	29.13	69.90-77.50	24.61-35.46	5.45	1.56	0.98	Chang7-2	
qTL3b	3	Bin3_1276	180.16	161.99-171.17	177.41-180.82	7.03	2.91	-0.98	Zheng58	
qTL4	4	Bin4_193	17.61	44.36-53.19	13.88-18.58	4.14	1.83	-0.79	Zheng58	
qTL5	5	Bin5_1719	188.80	207.77-226.9	183.70-189.20	7.37	3.65	-1.01	Zheng58	
qTL6	6	Bin6_1229	163.42	184.61-189.75	163.10-164.15	10.58	3.90	-1.05	Zheng58	
qTL8a	8	Bin8_1056	139.32	159.46-165.08	136.54-142.27	4.68	1.90	-0.55	Zheng58	
qTL8b	8	Bin8_1285	161.08	209.02-225.61	160.76-166.43	4.96	1.40	-0.57	Zheng58	
qTL8c	8	Bin8_1473	171.26	240.09-251.06	170.13-171.99	4.41	1.04	-0.63	Zheng58	
qULN2	2	Bin2_1790	236.42	233.32-238.90	234.77-236.84	18.18	6.04	0.34	Chang7-2	zap1
qULN3a	3	Bin3_1245	178.17	160.95-168.89	176.73-179.69	4.66	7.71	-0.24	Zheng58	
qULN3b	3	Bin3_1375	187.67	181.30-186.24	186.19-188.27	8.60	3.25	-0.26	Zheng58	
qULN5	5	Bin5_1401	159.30	162.18-169.74	150.97-160.15	4.85	1.78	0.16	Chang7-2	
qULN7	7	Bin7_222	21.82	52.38-56.09	19.89-25.15	6.62	2.01	-0.19	Zheng58	
qULN10	10	Bin10_84	6.10	27.49-36.7	4.94-6.92	6.23	2.20	0.19	Chang7-2	

Table S8. Markers used for verifying *qPH1a*.

Marker	Chr.	Pos.	Forward primer (5'–3')	Reverse primer (5'–3')
Indel_1-87	1	87.57	GAGGTGAACGCTTGGACGAT	GAACCTGGGTCCTCCTACGC
Indel_1-89	1	89.24	CTGTTCTGTTTCGTTTGGTGGC	GCCAAGGAAGCAGGCAACAG
Indel_1-91	1	91.18	GAACCACCACCGTCACCTCT	GTGCACTTGTAACGACCAAATAAA
Indel_1-97	1	97.51	TTTCGATGATGACGGCAATG	ACTATTCTCCCAATTCCCAAACA
